# Current Developments of *N*-Heterocyclic Carbene Au(I)/Au(III) Complexes toward Cancer Treatment

**DOI:** 10.3390/biomedicines10061417

**Published:** 2022-06-15

**Authors:** Alexia Tialiou, Jiamin Chin, Bernhard K. Keppler, Michael R. Reithofer

**Affiliations:** 1 Institute of Inorganic Chemistry, Faculty of Chemistry, University of Vienna, Währinger Str. 42, 1090 Vienna, Austria; alexia.tialiou@univie.ac.at (A.T.); bernhard.keppler@univie.ac.at (B.K.K.); 2 Vienna Doctoral School in Chemistry (DoSChem), University of Vienna, Währinger Str. 42, 1090 Vienna, Austria; 3 Institute of Inorganic Chemistry—Functional Materials, Faculty of Chemistry, University of Vienna, Währinger Str. 42, 1090 Vienna, Austria; 4 Research Cluster “Translational Cancer Therapy Research”, University of Vienna and Medical University of Vienna, Währinger Str. 42, 1090 Vienna, Austria

**Keywords:** cancer, gold complexes, *N*-heterocyclic carbenes, NHC, DNA, G-quadruplex

## Abstract

Since their first discovery, *N*-heterocyclic carbenes have had a significant impact on organometallic chemistry. Due to their nature as strong σ-donor and π-acceptor ligands, they are exceptionally well suited to stabilize Au(I) and Au(III) complexes in biological environments. Over the last decade, the development of rationally designed NHCAu(I/III) complexes to specifically target DNA has led to a new “gold rush” in bioinorganic chemistry. This review aims to summarize the latest advances of NHCAu(I/III) complexes that are able to interact with DNA. Furthermore, the latest advancements on acyclic diamino carbene gold complexes with anticancer activity are presented as these typically overlooked NHC alternatives offer great additional design possibilities in the toolbox of carbene-stabilized gold complexes for targeted therapy.

## 1. Introduction

### 1.1. Retrospect in Use of Medicinal Gold

Gold, one of the most precious metals on earth [1], was considered a panacea for numerous diseases along the centuries [2]. There are plenty of references to its medical use dated as early as 2500 BC from China and India to the Arabic world and the area now-known as Europe [3]. A contributing factor in the exploration of gold chemistry were the alchemists and their pursuit for longevity [4]. The realization that *aqua regia* dissolves elemental gold led to the concomitant use of elemental gold and its compounds in medicine [5]. Thus, potable gold (*aurum potabile*) emerged, and it was used extensively as a youth elixir and a treatment for multifarious conditions [6].

**Figure 1:**
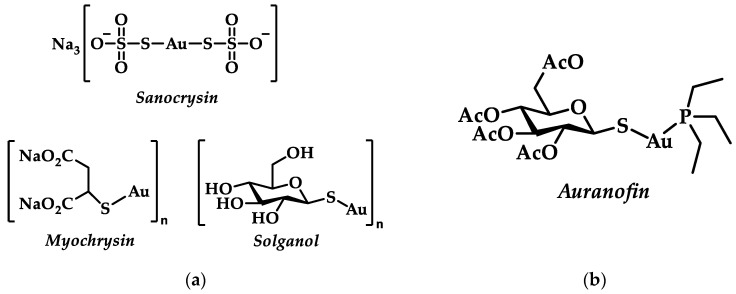
(**a**) Au(I) complexes used for the treatment of RA; (**b**) Chemical structure of orally-active drug, auranofin.

One of the first (13th c.) documented medical uses of gold is that of gold chloride, mentioned by R. Bacon, for the treatment of leprosy [1]. Later on, Paracelsus utilized gold in the cure of tuberculosis [7], while Li Shizhen composed (1578) the first review on gold drugs, containing the first systemic summary of Chinese medical records till then [8]. In the early 19th century, A. J. Chrestien and P. Figuier suggested a treatment against syphilis via the gold sodium chloride complex Na[AuCl_4_] known as *muriate of gold and soda* [6]. With Koch’s experiments of utilizing gold cyanide (K[AuCN_2_]) in bacterial cultures against *tubercle bacillus* in 1890, gold was in the vanguard toward tuberculosis treatments [9]. Robert Koch’s experiment can be seen as the beginning of gold molecular pharmacology and drug design [10]; during the “gold decade” (1925–1935), extensive research for less toxic but highly active Au(I) complexes was conducted, aided by the introduction of thiol ligands [10]. Compounds such as sodium aurothiosulfate (*Sanocrysin*), sodium aurothiomalate (*Myochrysin*), or aurothioglucose (*Solganol*) became popular (Figure 1a) [2]. After the rejection of gold-based drugs for tuberculosis treatment, it was Landré who first suggested their use for antiarthritic therapy based on a putative correlation with tuberculosis; however, this application was only later explored by Forestier [11]. Sodium aurothiomalate and aurothioglucose were exploited nevertheless in clinical use for rheumatoid arthritis (RA) [5,9].

Chrysotherapy is the use of gold drugs aiming at reducing inflammation and disease progression of RA [3,9]. Numerous adverse effects appeared due to intravenously dispensed drugs during chrysotherapy, with the most severe being related with multifarious dermatitis, bone marrow suppression, and nephrosis [3,12]. Apart from the already known intravenously used gold(I) thiolates, a new orally-active drug, auranofin, (Figure 1b)emerged [13,14]. Unlike the other polymeric water-soluble gold thiolates, auranofin, bears a phosphine ligand contributing to its lipophilic character [2,9,15]. However, long term use of auranofin resulted in side effects such as acute skin reactions (pruritus), stomatitis, gastrointestinal inflammation and chrysiasis corneae, thus limiting further applications [14]. Nevertheless, auranofin was approved by the FDA for the treatment of RA in 1985 [12].

The repurposing of the aforementioned gold drugs for anticancer treatment triggered the ongoing research towards Au(I) and Au(III) complexes in this field, offering a relatively affordable and efficient way toward novel drug discovery. A rational design of the coordination sphere of Au(I) compounds is utilized to control stability, lipophilicity, and the binding properties of these complexes [16]. Au(III) complexes, though considered as a promising alternative to platinum based anticancer drugs due to their similar d^8^ electronic configuration and structural conformation, face several challenges such as kinetic instability and photosensitivity, which slowed down the development of highly active Au(III) complexes [2,17]. Furthermore, careful ligand design is needed as Au(III) complexes can be reduced to Au(I) and Au(0) in a biological setting, such as by reacting with thiols and methionine residues in proteins and peptides [5,18,19]. A possible solution to the aforementioned problems is the use of strong donor ligands such as *N*-heterocyclic carbenes (NHCs), which help to stabilize Au(III), thus reducing the likelihood of unwanted ligand exchange reactions [20,21]. Similarly, an increase in stability can also be achieved using chelated ligands with softer, more polarizable donor atoms such as [N,N-], [C,N-], [C,N,N-], or [N,C,N-] [20,22]. Reports of Au(I) anticancer complex candidates with diverse ligands such as thiolates, phosphates, NHCs, alkynyl, and thiourea can be found in various reviews [22,23,24]. However, the scope of this present review mainly focuses on *N*-heterocyclic carbene bearing compounds.

### 1.2. Gold Chemistry

**Figure 2:**
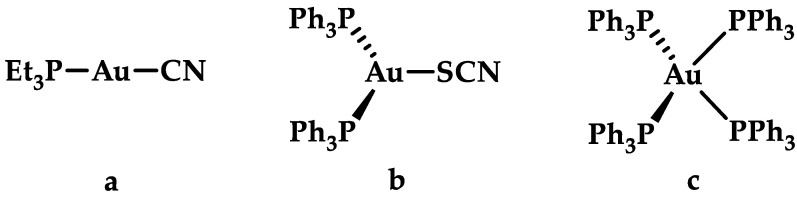
Different coordination geometries of Au(I) complexes with coordination number (**a**) 2, linear; (**b**) 3, trigonal planar; and (**c**) 4, tetrahedral.

The oxidation states of gold may vary from −1 to +5, nevertheless under aqueous physiological conditions, the relevant gold oxidation states encountered are +3, +1, and 0 [2,9]. Due to the high propensity of Au(I) salts disproportionate to Au(III) and Au(0), Au(I) salts are not stable in a biological setting, necessitating the need of stabilizing Au(I) through ligands. Typically, ligands capable of stabilizing Au(I) are either strong σ-donors or π-acceptor ligands, making *N*-heterocyclic carbenes a popular choice. The electronic configuration of Au(I) is d^10^, and ligands do not contribute an additional crystal field stabilization energy. Thus, Au(I) favors three different coordination geometries: a linear two-coordination, a trigonal planar three-coordination, and a tetrahedral four-coordination (Figure 2) geometry [5,25,26]. Based on the hard-soft acid-base theory (HSAB), Au(I) is considered a “soft” metal ion that coordinates preferentially to soft ligands such as S- or P-donors rather than O- or N-donors [25,27], while d^8^ Au(III) complexes are more stable when coordinated with N-donors and/or chelating ligands [27]. Similar to Pt(II), the most prevalent coordination geometry of Au(III) is square planar [5].

Ligands play a crucial role in the coordination of metal complexes, influencing their biological activity [28]. With the emergence of *N*-heterocyclic carbenes there has been unprecedented interest toward their influence on Au(I)/Au(III) complexes used in cancer therapy.

### 1.3. Nitrogen (N)—Heterocyclic Carbenes

**Figure 3:**
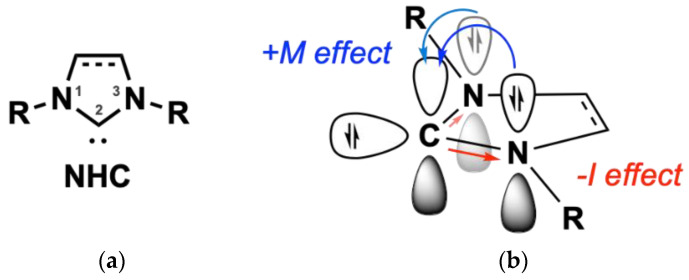
(**a**) Electronic stabilization of NHC of the free carbene electrons in C2 position (**b**) via σ-withdrawing and π-donating effects of the nitrogen heteroatoms. Reprinted with permission from Ref. [39]. Copyright (2021) Wiley-CVH.

Nitrogen (N)-Heterocyclic carbenes (NHCs) are a benchmark of contemporary organometallic chemistry. The first reported synthesis of an NHC dated back to 1961 by Schikora and Wanzlick, while in 1968 the first NHC-metal complexes were reported by Öfele and Wanzlick [29,30,31]. NHCs are suitable ligands for transition metals; their intrinsic σ-donor ability, owing to the free electrons of the heterocyclic ring, makes them nucleophilic toward metallic species (Figure 3a) [32]. The nitrogen atoms of the heterocyclic ring further electronically stabilize the molecule, while sterically bulky moieties connected to these nitrogen atoms contribute to kinetic stabilization (Figure 3b) [32]. In the case of gold, NHC coordination is enhanced due to relativistic effects [33]. The sp^2^ hybridization of the free electrons in NHCs interact with the sd-hybridized orbitals of the Au, thus contributing to a stronger NHC-metal bond [34]. The structural versatility of such ligands drew significant interest, particularly for homogenous catalytic applications as a promising replacement for phosphine ligands [35], e.g., in the generation of second generation Grubbs catalyst [36]. NHCs show similar coordination characteristics with phosphine ligands, proven by spectroscopic studies; however, their higher thermal and oxidative stability result in thermodynamically stronger metal-ligand bonds, enhancing the stability of their complexes to oxygen and water over phosphines [37,38]. NHCs can be synthesized quite easily based on a variety of different starting scaffold molecules such as imidazole, pyrazole, and triazole, and they are prone to numerous structural modifications [37]. This flexibility impacts their ability to coordinate with biomolecules (e.g., DNA and proteins) [16]. All the aforementioned characteristics, and especially the stability provided by NHC ligands, comprise vital features in the design of anticancer metal complexes, allowing an efficient delivery and transport to cancer cells.

From a ligand design perspective, NHC metal complexes are versatile as they can be easily tuned by installing a wide variety of the wingtip R-groups [16,40], significantly contributing to the ongoing search of NHC-based drugs. Especially gold-based NHC complexes with biological activity have been heavily investigated to date. The cellular and molecular targets of NHC Au(I)/(III) compounds vary from DNA and mitochondria damage and/or intervention to inhibition of the cell cycle, proteasomes and specific kinases, leading to apoptosis [27]. Besides the aforementioned target molecules, several studies underline their activity against other enzymes like cysteine proteases [41], aquaporins [42], protein tyrosine phosphatase [43], caspases [44,45], apoptosis inducing factor, and zinc finger [46]. As this review mainly addresses NHC Au(I)/(III) complexes for anticancer treatment, we refer the reader to additional review articles addressing the antimicrobial [47,48], antimalarial [49], anti-inflammatory [50], antioxidant [50,51], antileishmanial [50,52] and antiparasitic [53] activity of gold NHC complexes.

### 1.4. Cancer

Cancer refers to the erratic and the uncontrolled division of cells and their growth and/or spread to adjacent or remote organs and tissues [54]. The first recorded evidence of cancer can be dated back to Egypt in 3000 BC, according to Edwin Smith’s papyrus [55]. Hippocrates named it cancer due to the uncontrollable growth that resembles the movement of a crab; a name derived from the Greek word for crab (καρκίνος) [55,56].

The magnitude of cancer-related incidents worldwide makes cancer either the first or the second major cause of death, with the most frequent types being prostate and lung cancers for men and breast and cervical cancers for women [57]. Statistically, based on the results of 2020 (19.3 million cases), there is an estimation of an acute increase (47% rise) of cancer cases by 2040 at about 28.4 million [57]. However, the aforementioned prediction does not consider the impact of the COVID-19 pandemic. Recently Schüz et al. correlated the severe acute respiratory syndrome coronavirus type 2 (SARS-CoV-2) ramifications with the likelihood of acute lymphoblastic leukemia (ALL) on children, giving a new urgency to cancer research [58].

**Figure 4:**
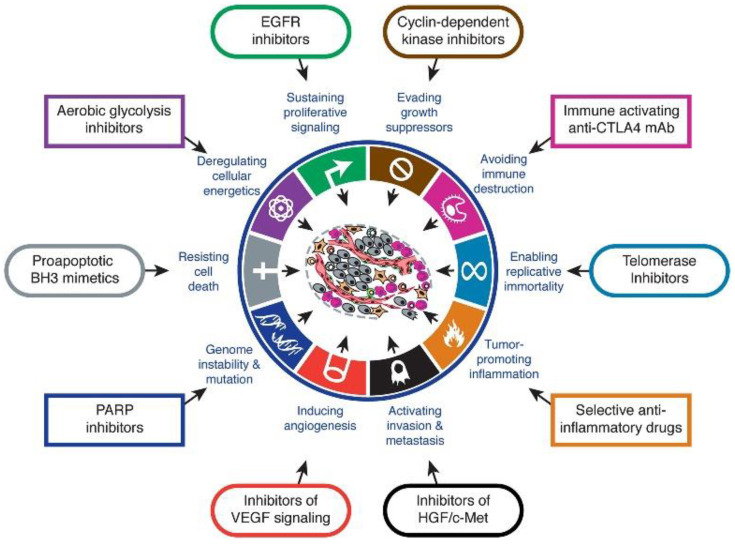
The hallmarks of cancer and their therapeutic targets. Reprinted with permission from Ref [65]. Copyright (2011) Elsevier Inc.

Carcinogenesis is a multistep process encompassing genetic alterations that are the outcome of cellular defects [59]. The biological characteristics acquired among neoplastic cells during their development in different steps of human cancer, also known as hallmarks of cancer, aid their survival, proliferation, and dissemination (Figure 4) [60]. As such, these genetic factors in combination with environmental factors enhance the transformation of normal cells into those that are cancerous [61]. Furthermore, tumorigenesis can be also triggered by viruses (e.g., HPV), initiating complications into the cell cycle via oncogene insertion or faulty stimulation of transcription of these oncogenes [62,63]. More in depth information regarding the cancer mechanism and the role of Au(I)/Au(III) carbene complexes in cancer can be found in the recent reviews of van der Westhuizen et al., Zou et al., or Mora et al. [22,24,64].

Currently there are three major treatment strategies for cancer: surgery, chemotherapy, and radiotherapy. These methods are usually used in combination with the aim to increase the success rate of any treatment [66]. Chemotherapy emerged in the 1940s as a promising combinatorial treatment with surgery against lymphomas, using nitrogen mustards [66,67]. For the next two decades, the already popular antibiotic drugs doxorubicin and bleomycin were used in cancer treatment targeting malignant cells [68,69]. Only in 1980 did G-quadruplex (see Section 2.1) structures emerge as a possible drug target, suggesting protein interactions with these structures may lead to possible regulatory effects in telomerase activity [70,71]. At about the same period, drugs such as topotecan, mitoxantrone, or etoposide were clinically tested for their activity against topoisomerases I and II (enzyme inactivators) [69]. Since then, drugs developed against malignant cell proliferation prolonged the life expectancy of cancer patients [66]. However, the high mutational probability of tumor cells and the diversity in their genetic alterations result in poor selectivity and the buildup of resistance [72,73].

In this review, we focus on DNA as a cancer drug target. Development of neoplasms is inextricably bound to the crucial role of DNA in various functions such as cell division (mitosis), gene transcription, and transduction of genetic material [59,62]. Conventional anticancer drugs aim to directly interfere with and disrupt the function of DNA, while others inhibit enzymes participating in DNA synthesis [61]. Our prime focus is on Au(I/III) carbene complexes interacting with the secondary structure of DNA stabilizing G-quadruplex (G4) systems (Section 2.1) or acting as intercalators.

## 2. DNA as a Target Molecule

Medicinal inorganic chemistry thoroughly explores the impact and the use of more than 20 metals that play a vital role in our body’s biochemical processes [74]. Platinum (Pt), ruthenium [75], gallium [76,77] and gold attracted more attention throughout the years as promising anticancer metallodrugs [78]. The antiproliferative properties of cisplatin, cis-[Pt^II^(NH_3_)_2_Cl_2_], were serendipitously discovered in 1965 by B. Rosenberg, and it received FDA approval in 1978 for IV treatment of testicular, ovarian, and bladder cancer [79]. This early success triggered an unprecedented interest in metal complexes as anticancer agents. Extensive research was conducted on cisplatin and its mechanism of action [61], the severe side effects nevertheless posed a dire need of developing equally effective and less toxic analogues such as carboplatin or oxaliplatin [79]. Carboplatin was approved by the FDA in 1989 for the treatment of advanced ovarian cancer [80], while oxaliplatin is among the first effective antimetastatic drugs for colorectal cancer and forms DNA adducts leading to apoptosis [81].

The extended research on platinum-based drugs has resulted in several other similar drugs which are able to interact with DNA either via intercalation or via groove-binding mode [82,83]. Such structural modifications to the shape of DNA hinders the effective repair of the genome leading to cell death (e.g., when targeting cancer cells) [84]. A well-known example is the mode of action of cisplatin; cisplatin preferably binds among guanine nucleobases (intrastrand binding) bending and unwinding the DNA helix [85,86]. Based on the same idea of distortion of DNA, metal complexes bearing intercalative or partially intercalative ligands (e.g., planar aromatic moieties with π-π stacking ability) have been extensively explored [83,87]. Au(I/III) complexes bearing various NHC ligands and/ or other intercalative ligands have been in the spotlight in the last decade, suggesting an alternative to cancer treatment [22,46,88,89,90,91].

### 2.1. G-Quadruplex Systems and Telomeric DNA

**Figure 5:**
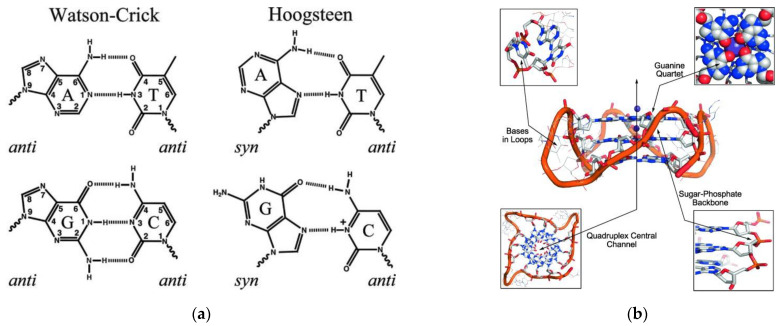
(**a**) Base-pair interactions. Reprinted with permission from Ref. [96]. Copyright (2013) Wiley Periodicals, Inc., Hoboken, NJ, USA; (**b**) structural features and targeted sites of a G-quadruplex. Reprinted with permission from Ref. [97]. Copyright (2010) Wiley—VCH Verlag GmbH & Co. KGaA, Weinheim, Germany.

One of the most famous self-assembled biopolymers in nature is DNA. Both DNA and RNA can adopt various secondary conformations, such as double helix, stem loop or pseudoknot [92,93]. Base-pair interactions among nucleobases are driven mostly by hydrogen bonds, however, both π-π stacking and hydrophobic forces implement a key role in stabilization of the structure [94]. Apart from the complementary Watson–Crick base-pair interactions, there are numerous base-pairing motifs that involve hydrogen bonds between the common nucleobases such as Hoogsteen base pairs (Figure 5a) [94]. Interactions between positively charged ions and the O-6 lone-pair electrons of each guanine stabilizes a planar array in which guanines are mutually bonded by Hoogsteen hydrogen base-pairing (Figure 5b) [95].

**Figure 6:**
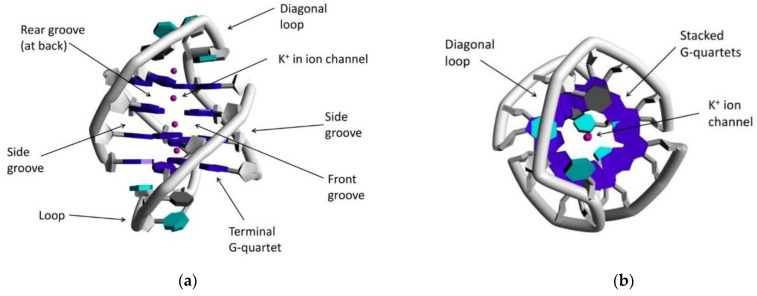
(**a**) Representation of G-quadruplex system with highlighted and labeled parts (PDB code 1JPQ); (**b**) view of the same quadruplex from different angle. Adapted with permission from Ref. [102]. Copyright (2016) American Chemical Society.

G-Quartet is a hydrogen-bonded “cyclic” tetramer based on guanine’s self-assembly. The four G residues that form a tetrad have a sugar–phosphate backbone interacting through Hoogsteen-type hydrogen bonds [98]. G-Quadruplex (G4), a non-canonical nucleic acid secondary structure, is comprised of stacked G-quartets on top of each other through π-system interactions leading to four-stranded guanine rich column-like superstructures stabilizing by coordinated monovalent cations K^+^ and Na^+^ ((Figure 6) [99]. Based on the polarity of DNA strands, G4 structures can be categorized as antiparallel-, hybrid-, or parallel-type G4 DNA [100,101] and are found in telomeric DNA, and other regulatory regions such as replication origins or in promoter regions of oncogenes [101,102]. Telomeric regions protect chromosomes from degradation and faulty/circumventing repair activities [103]. By every cell division, telomeric DNA grows shorter, gradually leading the cell to senescence; thus, the role of the enzyme telomerase is to maintain the length of telomeres to avoid cell apoptosis [59,103]. The role of G4 in cancer biology and its interaction with metal complexes has been thoroughly examined in the last decade [95], with one anti-cancer strategy being G4 stabilization to prevent cell division. In 1999, square planar metal complexes (porphyrins) acting as G4 binding stabilizers was first reported [97,104,105]. There has been continuous investigation in this direction, focusing on organic or metal-based compounds to stabilize G4 systems such as Schiff-based metal complexes (Ni^2+^, Zn^2+^, Pt^2+^ etc.) or organometallic Au(I) compounds with NHC ligands [106].

**Figure 7:**
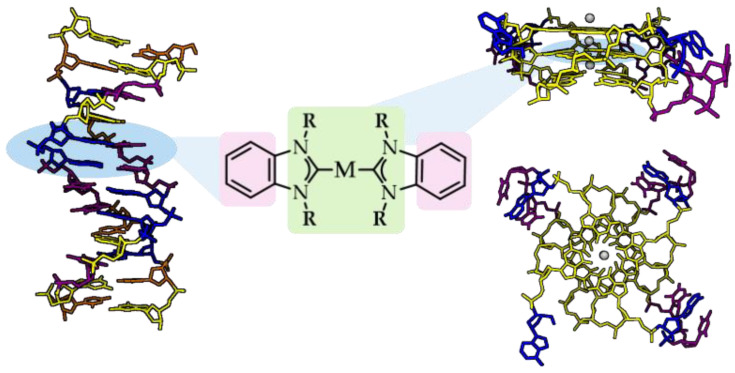
Rational design of ligand candidates intercalating with dsDNA (PDB code 1D28) and G-quadruplex motifs (PDB code 1KF1) via π-stacking of planar aromatic moieties (pink) and/or contributing R wingtips (light green). Created with BioRender.com.

In general, organometallic NHC complexes are not highly regarded for their binding affinity towards nucleic acids; nevertheless, their rational design to form aromatic/ planar moieties can impact their reactivity with canonical and non-canonical DNA secondary structures [89,107]. Rationally designed quadruplex-intercalating ligands should meet certain rules, such as possessing: (1) a flat and monocationic molecular structure capable of interacting with the negatively charged DNA; (2) one or ideally two antidiametric aromatic systems prone to interacting with guanines via π-stacking; (3) side chain water-solubilizing ligands, and (4) a metal center that can non-covalently interact with the carbonyl edges of the G-quartet over the central cation channel (Figure 7) [108,109].

### 2.2. Gold Targeted Biomolecules, Biodistribution, and Gold Prodrugs

Focusing on the unique chemistry of gold, scientists developed gold-based compounds against proteins, including enzymes and transport proteins. Thioredoxin reductase (TrxR) [110]; cysteine proteases [111]; cathepsins B, K, and S; and protein tyrosine phosphatases (PTPs) are some of the target molecules correlated with the high affinity of gold for cysteine and selenocysteine residues [22].

**Figure 8:**
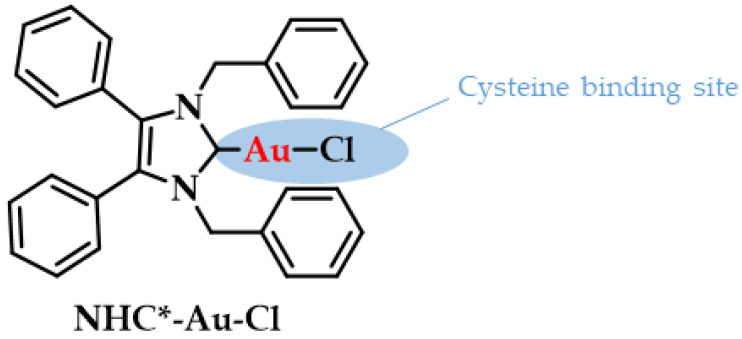
Chemical structure of NHC*-Au-Cl highlighting the albumin binding site. Adapted with permission from Ref. [117]. Copyright (2018) Wiley-VCHVerlag GmbH & Co. KGaA, Weinheim.

The pharmacokinetic profile of gold compounds are mainly obtained based on the measurement of the concentration of elemental gold inside the body [1]. For example, when gold compounds such as myochrysin or solganol are intramuscularly administered; the gold that is rapidly absorbed into the blood circulation can be found after 6 days either at about 75% excreted via kidneys in urine and 25% via bile into the feces (injectable gold salts). However, when an Au(I) drug is orally administered (auranofin) after crossing the gastric barrier the excretion percentages are 15% (urine) and 85% (feces), respectively [112]. Overall, the dose that is eliminated from the body is estimated at 40% for injectable gold and 80% for orally administered. Within the bloodstream, gold is bound to serum albumins of blood plasma due to the high gold affinity of Cys-34 in albumins; 80% of it in case of auranofin and 95% for injectable gold salts, while the rest is bound to globulins or red blood cells [113,114,115]. Experiments with radiolabeled Auranofin indicate that only 15% to 33% of the dose reaches the bloodstream compared to the total of the administered gold [115,116]. A recent example of an albumin bound gold drug is the 1,3-dibenzyl-4,5-diphenyl-imidazol-2-ylidene gold(I) chloride (NHC*-Au-Cl, Figure 8), which can conjugate to the cysteine of human serum albumin (HSA) to potentiate its drug efficacy as a potent anticancer agent. HSA is a suitable means for drug-delivery (e.g., to tumors) due to its abundance in the blood, and it can contribute to in vivo applications by biodistribution and formation of functional protein conjugates [117].

As for Au(III), only limited information is available about the metabolism, biodistribution, and pharmacokinetics of Au(III). An unexpected biochemical pathway can take place, contributing to in vivo Au(III) formation; Au(III) may form in vivo in case of oxidation by hypochlorite or myeloperoxidase after the intravenous injection of an Au(I) drug [27]. A recent insight on radiolabeled Au(III)-NHC complexes’ biodistribution was given by Salassa et al.: [^124^I]Au(III) complexes rapidly distributed through the blood stream and Au(III) accumulated in liver, kidneys, and lungs in higher concentrations comparing to brain, bladder and stomach; overtime, but not instantaneously, they reduced to Au(I) [51].

Pro-drugs are bioconverted in vivo by chemical or enzymatic metabolic processes to their active analogues affording (usually after structural rearrangement) active metabolites [118]. Gold compounds can function as pro-drugs by modulating their redox character and ligand exchange reactions, delivering and selectively activating them at the sites of inflammation and/or ailing cells. Biomolecular moieties such as thiols, imidazoles, and selenols are the coordination sites of the gold compounds after activation, usually via an alkylation mechanism [5,18,27]. Cellular uptake studies indicated that high amounts of NHC ligands and gold can be accumulated in the body if there is a presence of an intact Au-NHC moiety; cellular bioavailability though is negatively influenced by the proteins (serum components) of the medium due to the transformations that may happen to the gold complexes [119].

## 3. Recent Examples of NHC Au(I) and Au(III) Anticancer Drugs

### 3.1. General Synthetic Approaches to Obtain NHC Au(I/III) Complexes

**Figure 9:**
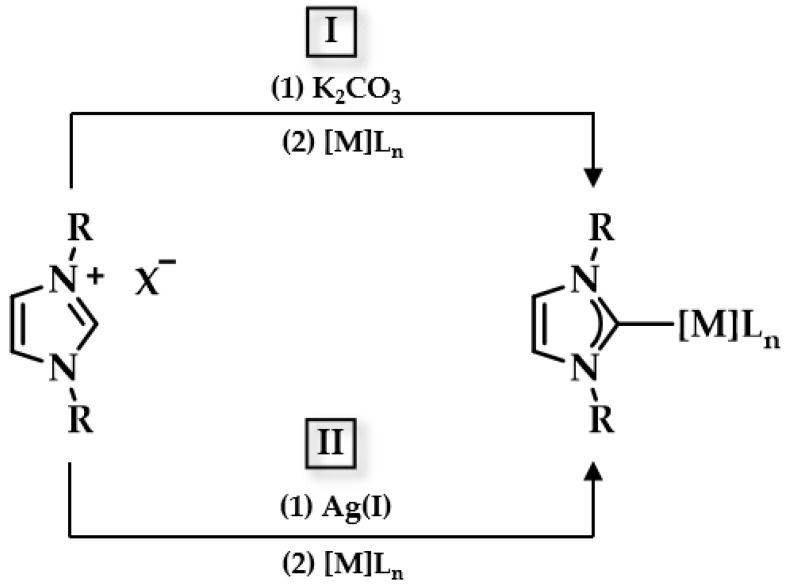
Synthesis routes towards NHC complexes **I** via mild base and **II** transmetalation with Ag_2_O.

Since the important role of NHCs in complex chemistry (see Section 1.3), many scientists associated their research with the synthesis of Au(I/III) carbene complexes. Lappert and coworkers were the first to report the formation of the NHC-Au(I) complex [120]. The synthesis of such a complex can be achieved via multiple routes [121,122,123]. Some commonly employed routes are a transmetalation reaction between NHC-Ag(I) and a Au(I) precursor such as chloro(dimethyl sulfide)gold(I) and the reaction of an Au (I) complex with free NHCs [35]. Lin and Wang were the first to explore the transmetalation route: benzimidazolium salts were treated with silver oxide (AgO_2_) under inert conditions, and the desired NHC-Au(I) complex was yielded after the successive addition of chloro(dimethyl sulfide)gold(I) in high yield (Figure 9) [124,125]. More recently, Nolan and coworkers established another approach toward the preparation of NHC-Au(I) complexes: a one pot reaction of Au(I) precursors reacting with imidazolium salts in the presence of K_2_CO_3_ affords NHC-Au(I) complexes in high yield, avoiding the use of dry solvents and inert conditions (Figure 9) [126].

**Figure 10:**
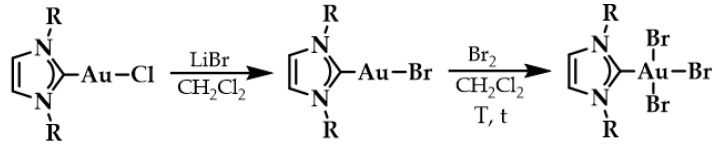
Synthesis of [AuBr_3_(NHC)] complexes.

NHC-Au(III) complexes on the other hand are commonly synthesized through the oxidation of its Au(I) precursor. A common approach was reported by Raubenheimer and coworkers in 1997, and ten years later a similar synthesis was described by Nolan et al. A direct oxidative addition reaction between a dihalogen (Cl_2_, Br_2_, I_2_) and a cationic bis(carbene) Au(I) complex was achieved in dichloromethane (Figure 10), affording the desired NHC-Au(III) complex [127,128]. One of the recent approaches involved simple reflux reactions among Au precursors bearing pincer ligands [Au(C^N^C)Cl] in the presence of NHC ligands under basic conditions [107,129]. Overall, there are several synthetic routes to synthesize Au(I/III) carbene complexes, and for a more detailed description we would like to refer to other reviews [35,122,130,131,132,133,134].

### 3.2. Gold(I) NHC Complexes

Starting with chrysotherapy, Au(I) compounds were employed against rheumatoid arthritis, thus research initially focused on gold compounds in the oxidation state +1 [3]. Auranofin, which was approved by the FDA for RA in 1985, posed a whole new perspective to “drug discovery,” after its repurposing to target cancer [12,135,136]. The increase of Au(I) complex stability which was achieved through the introduction of NHCs ligands significantly contributed to the development of novel Au(I) complexes, with high biological activity.

**Figure 11:**
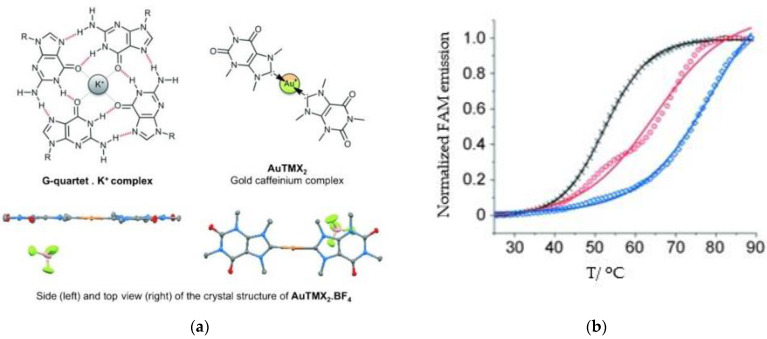
(**a**) Chemical structures of G-quartet (left) and [Au(9-methylcaffeine-8-ylidene)_2_]^+^ (**AuTMX_2_**) complex; (**b**) graph of FRET melting results of **AuTMX_2_**; 0.2 μM DNA with **AuTMX_2_** (red curve) and withthout any ligands (black curve). Adapted with permission from Ref. [108]. Copyright (2012) Wiley-VCHVerlag GmbH & Co. KGaA, Weinheim.

A leading example in the last decade of a rationally designed Au(I) complex which favors quadruplex-DNA over dsDNA is a nature-inspired planar Au(I)-NHC complex bearing two caffeine ligands, [Au(caffein-2-ylidene)_2_][BF_4_] (**AuTMX_2_**) (Figure 11a), which was developed by Cassini and coworkers. The compound was evaluated against four different DNA architectures (duplex and quadruplex DNA and three- and four-way DNA junctions). Further, competitive fluorescence resonance energy transfer (FRET) melting experiments were carried out confirming its ability to stabilize G4 DNA. FRET analysis is often used to show the stability or instability of duplex or G4 DNA by a ligand. With the formation of the G4, the fluorescence of the labelled 3′- and 5′-ends quenched, while due to the higher conformation perplexity of G4 the melting temperature increased (Figure 11b). Interestingly, the complex showed two-transition curves via FRET analysis (Figure 11b), indicating a multiple binding mode with the human telomeric quadruplex [108,137]. The non-covalent interaction between telomeric G4 and **AuTMX_2_** was confirmed some years later through a combined ESI MS and X-ray diffraction (XRD) analysis [138], while metadynamics simulations highlighted that π-stacking and electrostatic interactions play a crucial role in stabilizing the gold complex/G4 adduct [106].

**Figure 12:**
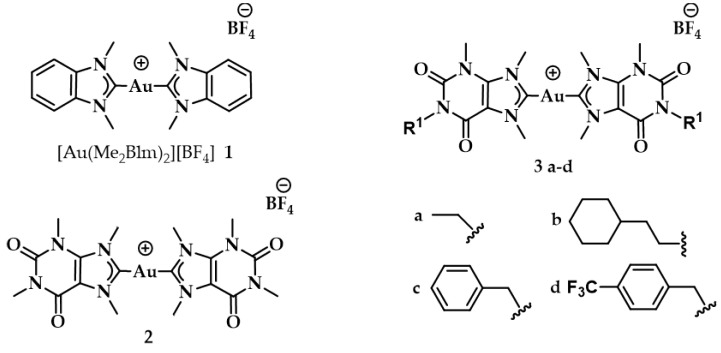
Chemical structures of Au(I) Benzimidazolylidene (Blm) and caffeine-based NHC complexes.

The same working group developed a series of caffeine-based and benzimidazolylidene NHC complexes (Figure 9) with antiproliferative activity and G4 stabilization character [139]. Of these complexes, compounds **1** and **2** (**AuTMX_2_**) showed high antiproliferative activity in vitro, however only **2** had very high selectivity, being toxic only toward malignant ovarian cells and cisplatin resistant analogues (A2780: IC_50_ = 16.2 ± 2.1 μM for **2**, IC_50_ = 5.2 ± 1.9 μM for cisplatin; A2780r: IC_50_ = 15.6 ± 2.7 μM for **2**, IC_50_ = 35 ± 7 μM for cisplatin); and it showed a slight toxicity toward non-malignant HEK-293T cells as well as in ex vivo healthy tissue. On the other hand, compound **1** had poor selectivity against cancerous and healthy cells. The coordination sphere of **1** enabled it to intercalate efficiently with duplex-DNA, while compound **2** with more sterically demanding ligands had a weaker affinity to duplex-DNA, and it was unable to intercalate. However, both complexes had a comparable effect on quadruplex stabilization where the steric hindrance was of little concern [139]. A similar group of compounds with a general formula [Au(N1-TBM)_2_]BF_4_ (N1-TBM=N1-substituted 9-methyltheobromin-8-ylidene) was synthesized by Bonsignore and coworkers (Figure 12) and compared with compound **2** for anticancer activity and G4 interaction. Whilst compound **2** was completely inactive against human melanoma (A375), ovarian (SKOV-3), and breast (MCF-7) cell lines, the bulkier substituted compounds **3b** and **3c** demonstrated an increase in cytotoxicity (**3b** A375: 8.8 ± 0.8 μM, SKOV-3: 13.0 ± 0.9 μM, MCF-7: 6.1 ± 0.8 μM; **3c** A375: 28 ± 3 μM, SKOV-3: 36 ± 4 μM, MCF-7: 18 ± 2 μM). However, the stabilization of the G4 conformation of hTelo and gene promoters of cKIT1 and hTERT was stronger with compound **2** when compared to complex **3b** and **3c**, respectively [140].

**Figure 13:**
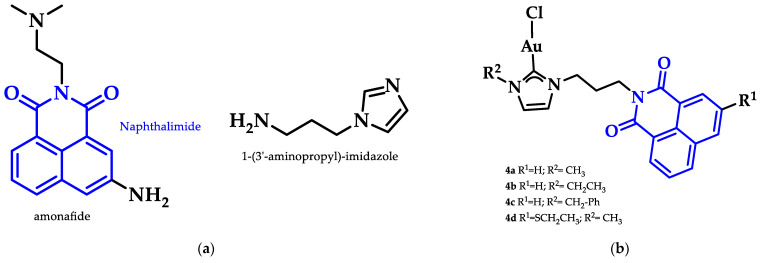
(**a**) Chemical structure of naphthalimide moiety and 1-(3′-aminopropyl)-imidazole ligands; (**b**) Chemical structures of series of Au(I)-NHC complexes with naphthimide moieties.

Ott et al. prepared a series of Au(I)-NHC positively charged complexes (Figure 13) as a side chain of a naphthalimide ligand. These complexes were tested for their cytotoxic activity against breast (MCF-7) and colon adenocarcinoma (HT-29) cancer cells. Complexes **4a**–**d** displayed enhanced cytotoxic activity, with **4d** having the lowest IC_50_ values (HT-29: 1.9 ± 0.3 μM, MCF-7: 2.1 ± 0.5 μM) compared to their imidazolium analogues against both cancer cell lines. The interaction of **4a**–**d** with DNA was evaluated with circular dichroism (CD) to assess the influence of R^1^ and R^2^ on the intercalation properties. Due to the positive charge of the complexes, they should interact with the negatively charged DNA backbone. The most effective intercalator of the Au(I) NHC complex series was **4a**. The R^2^ moiety of imidazole nitrogen better contributed to both cytotoxicity and G4 stabilization, when the methyl group was sterically less demanding [89].

**Figure 14:**
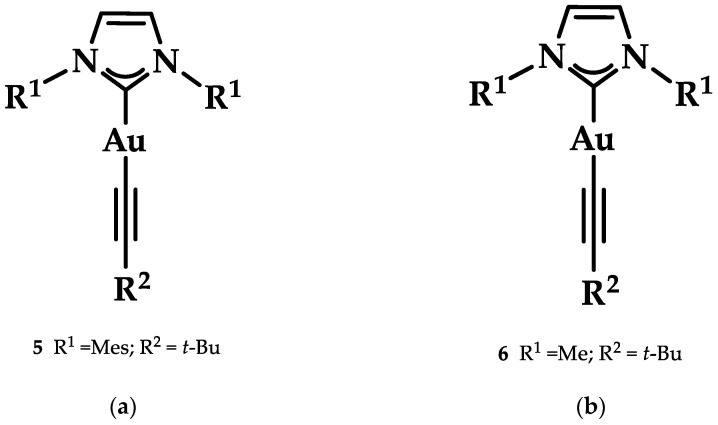
(**a**) Chemical structure of 1,3-dihydroimidazolylidene with mesitylene and *tert*-butylethynyl ligands; (**b**) chemical structure of benzimidazolylidene with methyl and *tert*-butylethynyl ligands.

Heteroleptic NHC Au(I)-alkynyl complexes and their analogues (Figure 14) were synthesized by Oberkofler et al. They were studied in vitro and compared to cisplatin against ovarian (SKOV-3), breast (MCF-7), and skin (A375) malignant cells. Compound **5** showed increased selectivity against melanoma A375 (EC_50_ = 3.4 ± 0.5 μM) over cisplatin (EC_50_ = 3.7 ± 0.9 μM) after 72 h of incubation. However, via FRET DNA melting analysis, only compound **6** showed a noteworthy stabilization of G4 in both human telomeric DNA (hTelo, 3.51 ± 0.08 °C) and the promoter of proto-oncogene tyrosine-protein kinase (c-KIT_1_) (7.00 ± 0.05 °C) [141].

**Figure 15:**
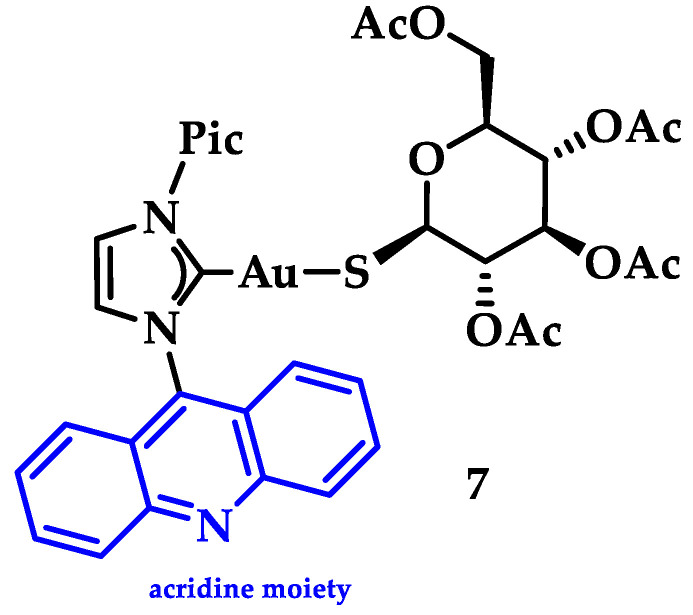
Chemical structure of acridine-based thiolate Au(I)-NHC.

Gimeno and coworkers synthesized a series of acridine-based thiolate Au(I)-NHC complexes, testing their cytotoxicity and biodistribution. Their antiproliferative activity was evaluated against lung (A549) and pancreatic (MiaPaca2) tumor cell lines. Here, the most cytotoxic complex of the series and a mimic of auranofin, **7** (Figure 15), (IC50 A549 = 13.0 ± 3.6 μM and MiaPaca2 = 3.4 ± 0.8 μM, cisplatin value 114.2 ± 9.1 and 76.5 ± 7.4 μM, respectively) is highlighted. Based on the reactivity motifof these compounds, as suggested by the authors, it is claimed that the most active moieties are the thiolate derivatives followed by those that are biscarbene and chloride. According to flow cytometry assays, a programmed cell death mechanism is suggested, while preliminary DNA interaction assays performed on plasmid pEYFP revealed an interaction of **7** with DNA. After a comparison with the acridine analogue that shows no DNA interaction, the observed nucleic acid affinity is attributed to the metal-NHC moiety [142].

### 3.3. Gold(III) NHC Complexes

Despite numerous examples of cytotoxic Au(III) complexes, their mechanism of action as well as specific cell targets are still mainly unknown. Nevertheless, the geometry of cisplatin inspired structurally analogous Au(III) complexes, which were expected to mimic cisplatin’s DNA targeting antitumor activity.

**Figure 16:**
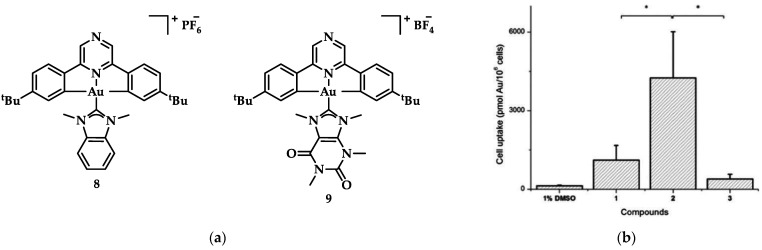
(**a**) Chemical structures of [(C^N^pz^^C)AuL]^+^ type complexes; (**b**) Cellular uptake graph where **2** corresponds to **8** and **3** to **9**. * *p* < 0.05. Adapted with permission from Ref. [143]. Copyright (2017) American Chemical Society.

One of the first reports of Au(III) compound bearing a pincer ligand interacting with G4 DNA was the [(C^N^pz^^C)AuL]^+^ with L = 1,3-dimethylbenzimidazol-2ylidene **8** (Figure 16), synthesized by Bertrand et al. [143]. Its G-quadruplex interaction was much stronger and dose-dependent compared to that of the caffeine analogue, [(C^N^pz^^C)AuL]^+^ (where L = 1,3,7,9-tetramethylxanthin-8-ylidene 9, Figure 16a) with hTelo, while there was also stability of the i-motif structure at varied pH. This distorted square planar complex (**8**) proved to have higher cytotoxic activity over a series of similar compounds and better cellular uptake (Figure 16b), with IC_50_ values at nanomolar scale comparing to the reference compound cisplatin against HL60 (0.31 ± 0.15 μM, cisplatin value 3.70 ± 0.25 μM), MCF-7 (0.56 ± 0.02 μM, cisplatin value 21.2 ± 3.9 μM), A549 (7.8 ± 1.3 μM, cisplatin value 33.7 ± 3.7 μM) and MRC-5 (1.4 ± 0.4 μM, cisplatin value 10.7 ± 3.0 μM) cancer lines [143].

**Figure 17:**
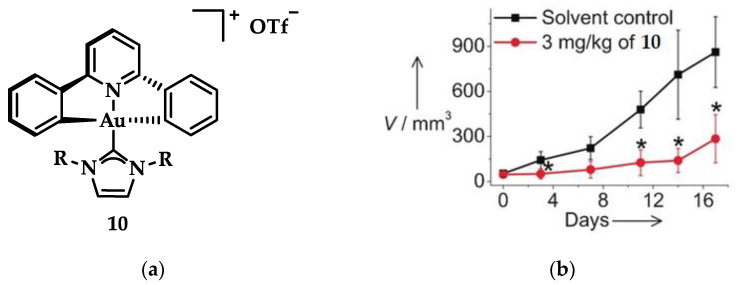
(**a**) Chemical structure of Au(III)-NHC pincer complex with R = *n*-butyl; (**b**) Antitumor activity in mice HeLa cells after treatment with 3 mg·kg^−1^ of **10**. * *p* < 0.05. Adapted with permission from Ref. [44]. Copyright (2017) Wiley-VCHVerlag GmbH&Co. KGaA, Weinheim.

Che et al. synthesized another series of Au(III) complexes with pincer ligands displaying strong and selective in vitro cytotoxicity and anticancer in vivo activity against mice carrying xenografts of human cervical and lung cancer. The mode of action of the Au(III)-NHC compound was verified using clickable photoaffinity probes that helped to identify the complexes’ interaction with intramolecular protein target molecules. One of the main targets, nucleophosmin, is an upregulating protein in cancer cells interacting with a positive regulator of tumor suppressor p53; this protein is involved in DNA replication and ribosome biogenesis. The micro dosing treatment of compound **10** (Figure 17a) in HeLa cells demonstrated a progressive formation of nucleophosmin monomers, increasing the expression of p53 protein levels. Che and coworkers observed that in vitro cytotoxicity against cervical (HeLa), colon (HCT116), and lung (NCI-H460) cancer cells enhanced with the increasing length of alkyl chain on NHC ligands. Antagonistic studies on HeLa cells with cisplatin and **10** indicated a greater reduction of spheroid growth by 10 (10: 70%, cisplatin: 30%). The same compound in in vivo studies using HeLa cells (Figure 17b) with 3 mg·kg^−1^ dosing showed an inhibition of growth at 71%, while NCI-H460 cells with the same dosage showed a 53% growth inhibition, without any adverse effects on body weight. Based on a series of compounds using 10 as a scaffold, an increased cytotoxicity against all the aforementioned cancer lines was observed with increasing alkyl chain length of R [44].

**Figure 18:**
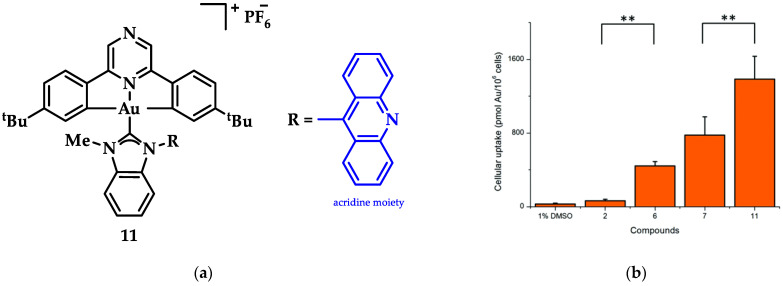
(**a**) Acridine-functionalized [(C^N^pz^^C)Au^III^(NHC)]^+^ complex; (**b**) Cellular uptake in MCF-7 cells (compound **11**). ** *p* < 0.005. Adapted with permission from Ref. [144]. Copyright (2018) The Royal Society of Chemistry.

An Au(III)-NHC complexes containing acridine moieties (11), was synthesized by Bochmann et al. and compared to analogues without a NHC ligand (Figure 18a). Uptake studies revealed a correlation between cytotoxicity and uptake, and compound 11 has the highest antiproliferative activity against breast (MCF-7) cancer cells (IC_50_ = 1.5 μM, cisplatin value 21.2 ± 3.9 μM). Based on studies of this series of compounds, it is indicated that the most cytotoxic complexes also have the highest cell uptake, thus demonstrating the importance of pharmacokinetics (Figure 18b). Acridine’s intercalating nature seems to contribute to the interaction with DNA, as **11** displays significant interaction with ds-DNA. However, comparative results of the other compounds highlight that the mechanism of action does not only rely on a simple intercalation of acridine, due to the poor DNA interaction of acridine itself [144].

**Figure 19:**
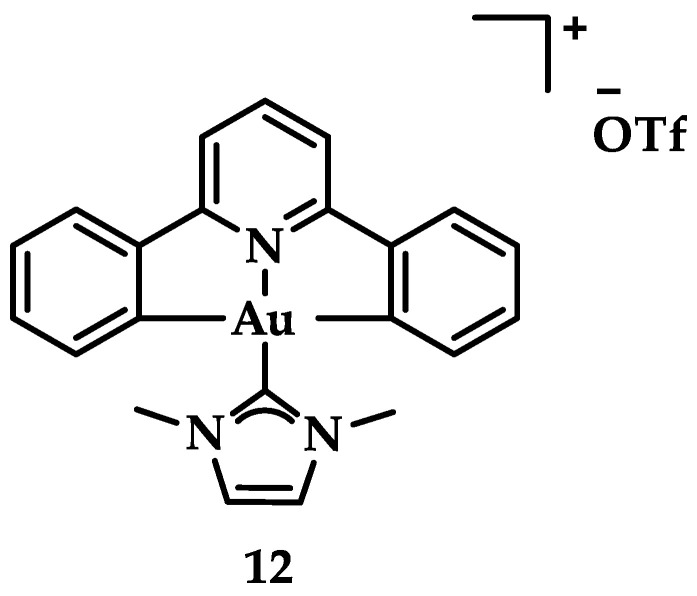
Chemical structure of complex [Au(C^N^N)(Ime)]PF_6_, with Ime = 1,3-dimethylimidazol-2-ylidene.

Neutral auxiliary NHC ligands were employed in synthesis of Au(III) complexes by Che et al. displaying a potential anticancer activity in vitro and in vivo. Complexes with the general type of [Au_n_(R–C^N^C)_n_(NHC)]^+^ (Figure 19) were studied for their interaction with DNA and a known intercalating compound, ethidium bromide (EB) [145]. The results indicated that the mode of binding is intercalation based on the binding constant K_b_ that demonstrates the magnitude of interaction with ctDNA (K_b_ = 5.4 × 10^5^ M^−1^) [146]. Complex **12** seems to hamper topoisomerase I (TopoI) mediated DNA relaxation. Further in vivo studies were also conducted on a nude mice model; in a 28-day period, a dosing of 10 mg·kg^−1^/week of **12** administered to mice resulted in 47% tumor suppression growth, without apparent toxic side-effects such as death or weight loss [107].

### 3.4. Hetero-Bimetallic Gold Containing NHC Complexes

**Figure 20:**
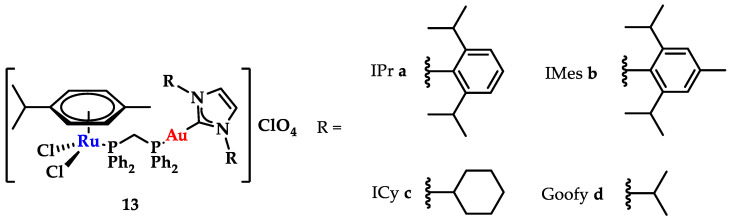
Heterobimetallic ruthenium-gold complexes [Ru(p-cymene)Cl_2_(μ-dppm)Au(NHC)]ClO_4_ [147].

NHCs show limited DNA interaction, unless rationally designed to target and interact with DNA quadruplexes [20]. However, through a combination with other metals, new avenues in drug design can be explored. Contel and coworkers synthesized a series of heterobimetallic Ru-Au complexes (Figure 20) with potent anticancer activity [147]. Based on screening against renal (Caki-1), colon (HCT 116 and DLD1), and non-tumorigenic kidney (HEK-293T) cell lines against cisplatin, heterobimetallic compounds **13a**–**d** indicated a high cytotoxicity against malignant cells, while they were less toxic toward healthy cells. Gel electrophoresis studies using plasmid DNA showed only a weak effect on DNA supercoiling, even when the compound concentration was increased, and a weak DNA interaction via a non-intercalative binding [147].

**Figure 21:**
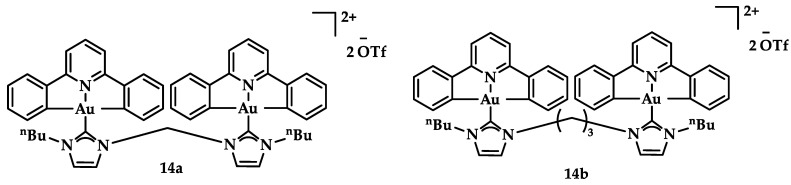
Chemical structures of dianionic **14a** and **14b**.

Dianionic [C^N^C]^2−^ auxiliary NHC ligands were employed in the synthesis of Au(III) complexes by Che et al., displaying a potential anticancer activity in vitro and in vivo. Compounds **14a** and **14b** (Figure 21) showed promising anticancer activity against hepatocellular carcinoma (HepG2) with IC_50_ value 7.9 ± 0.6 μM and 1.1 ± 0.1 μM, respectively, while **13b** displayed cytotoxicity against camptothecin-resistant cells (KB-CPT-100) with an IC_50_ value of 12 ± 1.3 μM; further, **14a** displayed low cytotoxicity against the non-cancerous lung fibroblast cells (CCD-19Lu) with a value > 100 μM [107].

**Figure 22:**
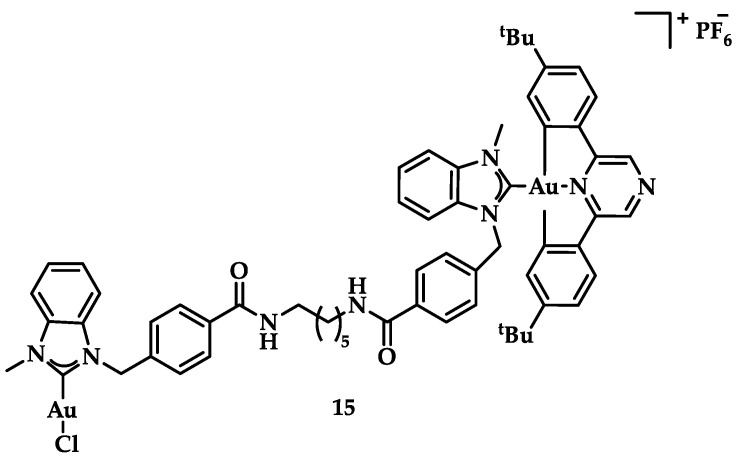
Chemical structure of bimetallic [(C^N^pz^^N)Au(NHC-C_6_-NHC-AuCl)]PF_6_ complex.

Cyclometalated bimetallic Au(III)/Au(I)-NHC complexes were synthesized and studied for their anticancer activity by Bochmann et al. Compound **15** (Figure 22) showed selectivity for breast cancer tumor cells MCF-7 and MDA-MB-231 (13.7 ± 2.2 μM and 9.2 ± 2.9 μM, respectively), compared to other tested cell lines [148].

**Figure 23:**
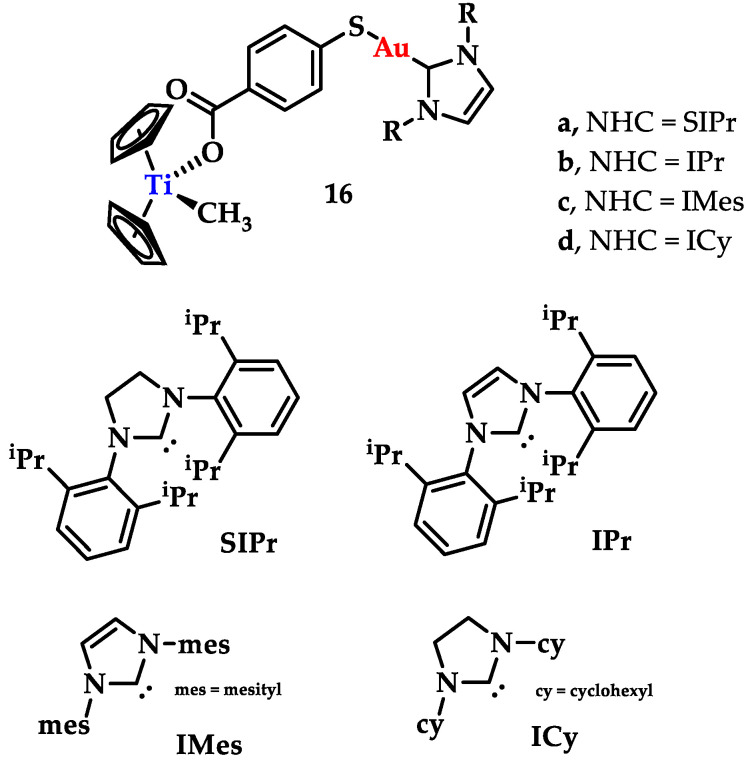
Chemical structure of bimetallic titanocene-gold-NHC complexes.

Contel and coworkers synthesized a series of titanocene-gold-NHC complexes [([(η^5^-C_5_H_5_)_2_TiMe(μ-mba)Au(NHC)] (NHC = SIPr (**16a**), IPr (**16b**), IMes (**16c**), ICy (**16d**)) as potential antitumor agents (Figure 23). They were tested against renal, prostate, colon, and breast cancer lines and the non-tumorigenic embryonic kidney cell lines, while titanocene was used as a control. The comparison between the bimetallic complexes and their monomeric analogues indicated that, apart from the breast cancer lines, the bimetallic compounds displayed a higher cytotoxicity than their monomeric analogues. Their values are summarized in Table 1 [149].

### 3.5. Acyclic Gold Compounds as Promising Alternative Anticancer Agents

NHC gold carbene complexes have become state of the art over the last decade, with numerous applications in the medicinal field [150]. Acyclic carbene (ACC) ligands, on the other hand, present a promising alternative due to their synthetic versatility, and they provide a variety of methods to improve solubility, cell uptake, and selectivity of cytotoxicity [151,152,153]. Some of the first examples of this category are the acyclic carbene adducts of gold; however, their biological impact is as yet hardly explored [148,154].

**Figure 24:**
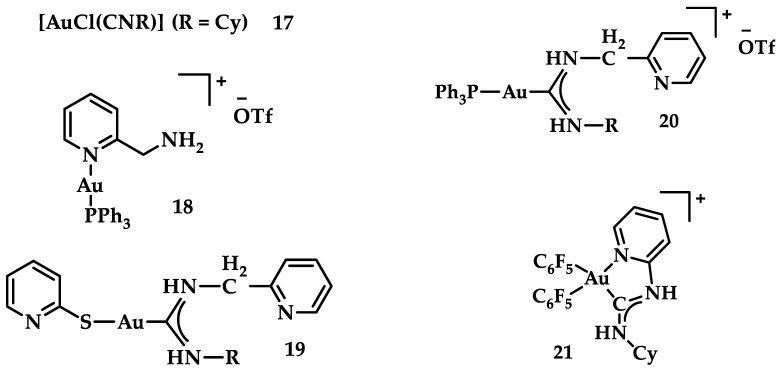
Chemical structures of Au(I) (**18**–**20**) and Au(III) (**21**) of neutral and/or cationic acyclic diamino carbenes.

A series of Au(I) and Au(III) of neutral or cationic acyclic diamino carbenes were synthesized by Gimeno et al. (Figure 24). These complexes were tested against leukemia T-cell (Jurkat), pancreatic (MiaPaca2), lung (A549), and breast (MDA-MB-231) cancer cell lines comparing to cisplatin reference compound. The more intense cytotoxic activity according to IC_50_ values was displayed by compound **17** against Jurkat (0.61 ± 0.2 μM), **18** against MiaPaca2 (5.30 ± 0.6 μM), **19** against A549 (9.85 ± 0.8 μM), and **20** and **21** against MDA-MB-231 (8.8 ± 1.6 μM and 16.0 ± 5.4 μM, respectively) [152].

**Figure 25:**
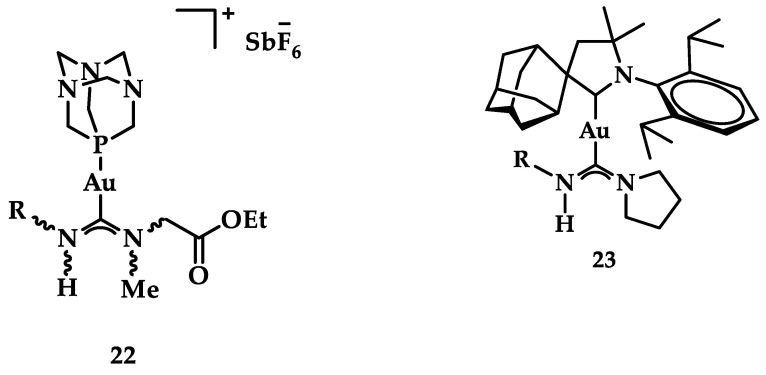
Chemical structure of mixed carbene compounds **22** and **23**.

A group of acyclic carbene (ACC) Au(I) complexes with chemical structures (ACC)AuCl, [(ACC)Au(PTA)]^+^ (PTA = triazaphosphaadamantane) and mixed-carbene compounds [(CAAC)Au(ACC)]^+^ (Figure 25), were synthesized by *Bochmann* et al. Apart from their X-ray diffraction characterization and redox properties, the compounds were studied for their antitumor activity against breast (MCF-7) and lung (A549) adenocarcinoma epithelial cell lines. All of the compounds except for **22** demonstrated strong antiproliferative activity against MCF-7 cancer cells at high concentrations (100 μM), while in the case of A549 cell line, only four of the compounds did so. The most promising complex is the mixed-carbene **23**, which shows good inhibition performance against both cell lines at two concentrations (10 and 100 μM). Equally impressive are also the IC_50_ values against the relevant cancer cell lines (IC_50_ = 0.10 ± 0.01; 2.3 ± 0.9 and 6.1 ± 0.3 μM against HL60, MCF-7 and A549 cells, respectively) [151].

## 4. Conclusions

The biomedical use of gold has come very far over the last decades. Significant focus has been placed on the rational design of NHC-Au(I/III) complexes, which interact with DNA so as to unlock novel, highly selective anticancer drugs. Although the current progress is very promising (see Table 2 for an overview of compounds), NHC-Au(I/III) complexes are still in their infancy when compared to the well-established platinum based anticancer counterparts, as can be seen from the lack of clinical trials of NHC-Au(I/III) drugs. The recent focus on G4-DNA targeted Au(I/III) complexes has however paved the way to understanding the contribution of G4 stabilization-induced DNA damage in cancer treatment; nevertheless, only a handful of information from DNA damaging agents (G4 known ligands) is available through pre-clinical and clinical data, leaving the mechanism of action still mostly unknown [109]. However, NHCs offer significant opportunities in drug design as important pharmacological properties such as complex stability or lipophilicity can be fine-tuned through ligand design. In many cases, there was a profound structure–activity correlation; however, we cannot generalize on the impact of steric bulk on NHC ligands as there is a separate direct correlation within each specific series of compounds reported. Furthermore, the use of ADC offers an underexplored area with interesting potential for the rational design of Au anticancer drugs, where the mode of action might defer from current NHC compounds. Future development of Au(I/III) should take lessons learned from the development of targeted drugs, and apply concepts such as the use of nano formulations, conjugation to targeting entities or bioconjugation to NHC-Au(I/III) complexes, which may lead to exciting new discoveries.

## Figures and Tables

**Table 1:** IC_50_ values (μM) in human cell lines.

Compound	PC3	DU-145	Caki-1	DLD1	MDA-MB-231	HEK-293T
**titanocene**	58.1 ± 11.2	55.2± 7.9	29.4 ± 4.2	56.2 ± 9.8	18.0 ± 3.6	>200
**16a**	9.8 ± 2.2	11.8 ± 3.0	21.0 ± 1.9	13.9 ± 1.7	>100	58.8 ± 6.7
**16b**	10.3 ± 2.8	18.9 ± 3.0	51.5 ± 3.7	30.4 ± 4.1	>100	>100
**16c**	17.1 ± 2.9	13.76 ± 2.7	29.11 ± 4.1	19.9 ± 4.1	>100	69.7 ± 9.9
**16d**	11.8 ± 1.6	16.7 ± 2	42.9 ± 5.8	21.5 ± 2.0	>100	77.1 ± 9.1

**Table 2:** Summary of IC_50_ values of complexes.

Gold(I) NHC Complexes						
No.	Compounds	IC_50_ Values (μM)				Ref.
**2**	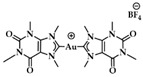	A2780 16.2 ± 2.1 (5.2 ± 1.9, cisplatin)	A2780r 15.6 ± 2.7 (35 ± 7, cisplatin)			[139]
**3b**	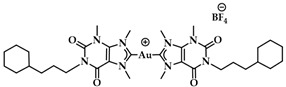	A375 8.8 ± 0.8	SKOV-3 13.0 ± 0.9	MCF-7 6.1 ± 0.8		[140]
**3c**	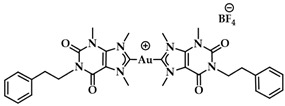	A375 28 ± 3	SKOV-3 36 ± 4	MCF-7 18 ± 2		[140]
**4d**	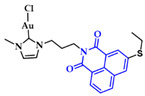	HT-29 1.9 ± 0.3	MCF-7 2.1 ± 0.5			[89]
**5**	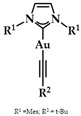	A375 3.4 ± 0.5 (3.7 ± 0.9, cisplatin)				[141]
**7**	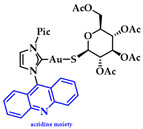	A549 13.0 ± 3.6 (114.2 ± 9.1, cisplatin)	MiaPaca2 3.4 ± 0.8 (76.5 ± 7.4, cisplatin)			[142]
**Gold(III) NHC Complexes**						
**8**	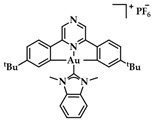	HL60 0.31 ± 0.15 (3.70 ± 0.25, cisplatin)	MCF-7 0.56 ± 0.02 (21.2 ± 3.9, cisplatin)	A549 7.8 ± 1.3 (33.7 ± 3.7, cisplatin)	MRC-5 1.4 ± 0.4 (10.7 ± 3.0, cisplatin)	[143]
**11**	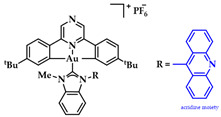	MCF-7 1.5 (21.2 ± 3.9, cisplatin)				[144]
**Hetero-Bimetallic Gold Containing NHC Complexes**						
**13b**	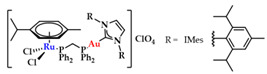	KB-CPT-100 12 ± 1.3				[147]
**14a**	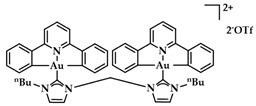	HepG2 7.9 ± 0.6				[107]
**14b**	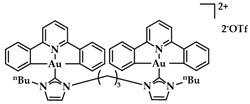	HepG2 1.1 ± 0.1				[107]
**15**	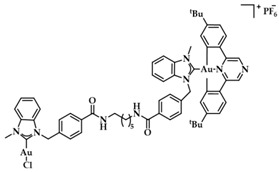	MCF-7 13.7 ± 2.2	MDA-MB-231 9.2 ± 2.9			[148]
**17**	**[AuCl(CNR)] (R = Cy)**	Jurkat 0.61 ± 0.2				[152]
**18**	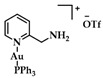	MiaPaca2 5.30 ± 0.6				[152]
**19**	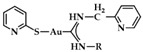	A549 9.85 ± 0.8				[152]
**20**	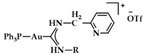	MDA-MB-231 8.8 ± 1.6				[152]
**21**	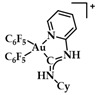	MDA-MB-231 16.0 ± 5.4				[152]
**23**	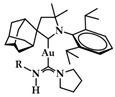	HL60 0.10 ± 0.01	MCF-7 2.3 ± 0.9	A549 6.1 ± 0.3		[151]
